# The development and reform of public health in China from 1949 to 2019

**DOI:** 10.1186/s12992-019-0486-6

**Published:** 2019-07-02

**Authors:** Li Wang, Zhihao Wang, Qinglian Ma, Guixia Fang, Jinxia Yang

**Affiliations:** 1 School of Health Services Management, Anhui Medical University, 81 Meishan Road, Hefei, 230032 Anhui People’s Republic of China; 2 The First Affiliated Hospital of USTC, Division of Life Sciences and Medicine, University of Science and Technology of China, 17 Lushan Road, Hefei, Anhui 230001 People’s Republic of China; 3 Department of Medical Law, Anhui Medical University, 81 Meishan Road, Hefei, 230032 Anhui People’s Republic of China

**Keywords:** Public health, Development, Reform, National Basic Public Health Service Program

## Abstract

**Background:**

Public health system plays a vital role in the development of health sector in China and protects the health of Chinese people. However, there are few comprehensive reviews and studies focusing on its evolution and reform. It is worthwhile to pay attention to the public health development in China, given that the history and structure of public health system have their own characteristics in China.

**Methods:**

The study is a retrospective review of the development public health over seven decades in China. It presents the findings from some national or provincial survey data, interviews with key informants, reviews of relevant published papers and policy contents.

**Results:**

This study identified four key stages that public health experienced in China: the initial stage centering on prevention, the stage of deviation with more attention to treatment but little to prevention, the recovery stage after SARS(Severe Acute Respiratory Syndromes) Crisis, and the new stage to an equitable and people-centered system. In the latest stage, the National Basic Public Health Service Program (NBPHSP) is implemented to respond the threat of noncommunicable diseases (NCDs) and has achieved some initial results, while there are still many challenges including service quality, poor integration among service items and IT system, lack of quality professionals and insufficient intersectoral endeavor.

**Discussion:**

There are unique Chinese wisdom and remarkable achievements as well as twists and turns on the development of China’s public health. Prevention-first, flexible structure of the system, multi-agency collaboration and mass mobilization and society participation are the main experience of public health in early stage. Despite twists and turns since 1980s, public health system in China shows substantial resilience which may be from the government’s continuous commitment to social development and people’s livelihoods and its flexible governance. In 2010s, in order to achieve the well-off society, Chinese government pays unprecedented attention to health sector, which bring a new wave of opportunities to public health such as remaining the NBPHSP for priority. The evolution and reform of China’s public health is based on its national condition, accumulates rich experience but also faces many common worldwide challenges. Getting this development and reform right is important to China’s social and economic development in future, and China’s experience in public health may provide many lessons for other countries.

**Conclusion:**

Public health in China needs to focus on prevention, strengthen multi-agency coordination mechanism, improve the quality of public health services in the future.

## Background

After its establishment in 1950s, China’s public health system remarkably eased the burden of infectious diseases as well as maternal, child and infant conditions [1, 2] therefore, reduced mortality rate and improved life expectancy greatly [3]. International organizations such as WHO once recognized it as a role model in health system for developing counties [4]. Afterwards, with the market-oriented reform in health sector [5], China’s public health system confronted huge challenges. Underfunded by the government [6], major public health service providers, namely, primary healthcare facilities and disease prevention control institutions, plunged into difficulties in market competition [7], had to support themselves with charged services, and compromised on equity of public health services [8].

Meanwhile, with economic and social development, the disease spectrum of Chinese has undergone noticeable changes, from infectious diseases to dual burden of the infectious diseases and non-communicable diseases (NCDs) [9–11] Therefore, on the one hand, control of conventional infectious diseases is still challenging. Some almost eliminated ones appear again. For example, in recent years, incidence and mortality rate of tuberculosis (TB) have rebounded [12]. There are also cases with meningitis, mumps, rubella, or measles now and then, and sometimes even endemic outbreaks [13–18]. Meanwhile, emerging communicable diseases also loom up, 1 every 1–2 years on average [19]. Continuous efforts such as vaccination, disease surveillance and so on are still required to control infectious diseases. On the other hand, the system also needs to manage a huge number of NCD patients, including 160–170 million hypertensive patients, over 100 million with hyperlipidemia, 92.4 million with diabetes, 70 million to 200 million overweight or obese people, and 120 million with fatty liver [20]. To address the “dual burden”, the “National Basic Public Health Service Program” (NBPHSP) was introduced in 2009 when the new round of healthcare reform was launched [21], and was set as one of important strategies to achieve the goal of “Healthy China 2030 Strategy” . Financed by the government to meet the basic needs in public health, the NBPHSP includes both population-based and group-specific services (women, children, the elderly and NCD patients). As a long-term institutional arrangement for equal access to basic public health services, it has been the public health intervention strategy covering the biggest areas and the most beneficiaries in the past 70 years since the founding of the P.R.C. [22], which shows government’s unprecedented commitment to this area.

After making remarkable achievements and experiencing many twists and turns, China’s public health is striving for a more equitable and more people-centered direction. It plays a vital role in the development of health sector and protects the health of Chinese people. However, there are few comprehensive reviews and studies on its evolution and reform. Internationally, the latest literature was published on Lancet at the end of 2018 as an editorial article (one-page) which generally introduced the achievements and the challenges of China’s public health [3]. Existing studies are not updated to reflect new changes and tendency [23–25], or just focus on primary health care in general [21, 26]. China’s primary healthcare system provides both essential clinical care and public health service [21]. The two are closely related to each other in disease control and health promotion, and their integration is increasingly important. Nevertheless, it is worthwhile to analyze public health as a relative independent system, and study the unique role of public-health, population-wide and community-based disease prevention and control, health protection and health promotion [27].

The term “public health” is always vaguely defined [17, 28]. According to Winslow, a leading public health expert, public health is the science and art of preventing disease, prolonging life, and promoting health through the organized efforts and informed choices of society, organizations, public and private communities, and individuals [29]. U.S. [30, 31], UK, WHO [32] Australia [33], WHO/WPR [34] and other countries or organizations have identified the basic functions of public health or the scope of basic services that should be delivered by public health system respectively. Internationally, public health is composed of services in 3 categories: (1) population-based public health services, including vector control and population-wide health education; (2) individual-based preventive care, for instance, vaccination, premarital checkup and prenatal care; and (3) individual-based curative care against conditions affecting health of the public, such as TB and STD [27]. Public health is closely related to social and economic development, demographic structure, disease pattern and disease burden and existing administrative system. Those factors vary a lot among countries and regions. The paper will unfold the development of China’s public health sector comprehensively, by describing its policy evolution in the past 70 years, analyzing outcomes of those policy changes, and reviewing the lessons from success and failure.

On a conference in 2003, then Chinese Vice-premier Wu Yi said: Public health is to organize the joint efforts of the whole society to improve environmental sanitation, prevent and control infectious diseases and epidemics, develop good hygienic habits and civilized lifestyle, and deliver health care so as to prevent disease and enhance people’s health [35]. Based on the literature review on the definition of public health, Hao [19] thinks it should cover 11 areas, including prevention and control of infectious disease and non-communicable diseases (NCDs), intervention on unhealthy lifestyle, maternal and child health, control of environmental hazardous factors, mental health, control against injury and violence, food and drug safety, and other issues bearing on health of the public (e.g. endemics and safety of blood product). China’s public health system consists of disease control system, health supervision and enforcement system and public health emergency response system [36]. The above definitions reflect the basic content and structure of public health in China. In this paper, the discussion and analysis of public health in China is mainly around the relevant content and structure.

## Methods

The study is a retrospective review of the development of public health in China over seven decades. We focus on this topic for the following reasons: (1) the corresponding author serves as an advisor and expert of the National Health Commission of China, and the authors have undertaken studies to evaluate the implementation of the NBPHSP, (2) the first author and the corresponding author have convenient access to key informants who are knowable about China’s public health system.

It presents the finds from some national or provincial survey data, interviews with key informants, reviews of relevant published papers and policy contents. Data sources in the study are mainly:

1. Literature Review. Both Chinese and English literature were retrieved on PubMed, Web of Science, CNKI and WanFang Database with “China”, “public health”, “development” and “reform” as key words. Related policy notes and data were also collected from website of the National Health Commission (NHC), the WHO and the World Bank Database, etc. based on the above criteria.
2. Interviews with Key Insiders. In-depth interviews were conducted with policy makers, front-line workers and other relevant personnel who have been engaged in public health for many years. One management staff from each of the three institutions, namely, Department of Primary Care of NHC, the National Disease Prevention and Control Bureau of NHC, and the national-level CDC, was interviewed, as they have good knowledge about the development of China’s public health sector. In addition, we also conducted some interviews in Anhui Province. With a large number of agricultural populations, the province is representative in China and many healthcare reform measures are piloted there. We interviewed one management staff from the Division of Primary Health in provincial health authorities, and one from the provincial CDC. Moreover, the interviewees also include some implementers in a county. They are a management staff from county-level health bureau responsible for public health, a head and a public health doctor in a township health center (THC) and a village doctor. They know the practices at primary level. The interviews can supplement and cross-validate the literature and quantitative data. All of the interviewees received verbal informed consent.
3. National or provincial survey. Entrusted by the Department of Primary Health, NHC, the authors started to evaluate the results of the NBPHSP in 2018, and obtained some data, including share of the financings from national government in the total investment in the NBPHSP in 31 provinces (autonomous regions or municipalities directly under the Central Government) in 2016, their per capita funding levels respectively, and some indicators of the NBPHSP from 2009 to 2016, such as, coverage of health record, coverage of electronic health record (EHR), utilization rate of health record, rate of postpartum newborn home visit, coverage of health management among children aged 0~6, rate of registration in early pregnancy, coverage of postnatal visit, coverage of health management among the elderly, percentage of hypertensive patients under standardized management, percentage of type 2 diabetic patients under standardized management, and coverage of health management by Traditional Chinese Medicine (TCM). In addition, other relevant data were also available, including infant mortality rate and maternal mortality rate in some parts of the world, and the results of the Sixth National Health Literacy Survey in China. The data were analyzed in Microsoft Office Excel 2010 for Windows (Microsoft Corp., Redmond, Washington).

**Fig. 1:**
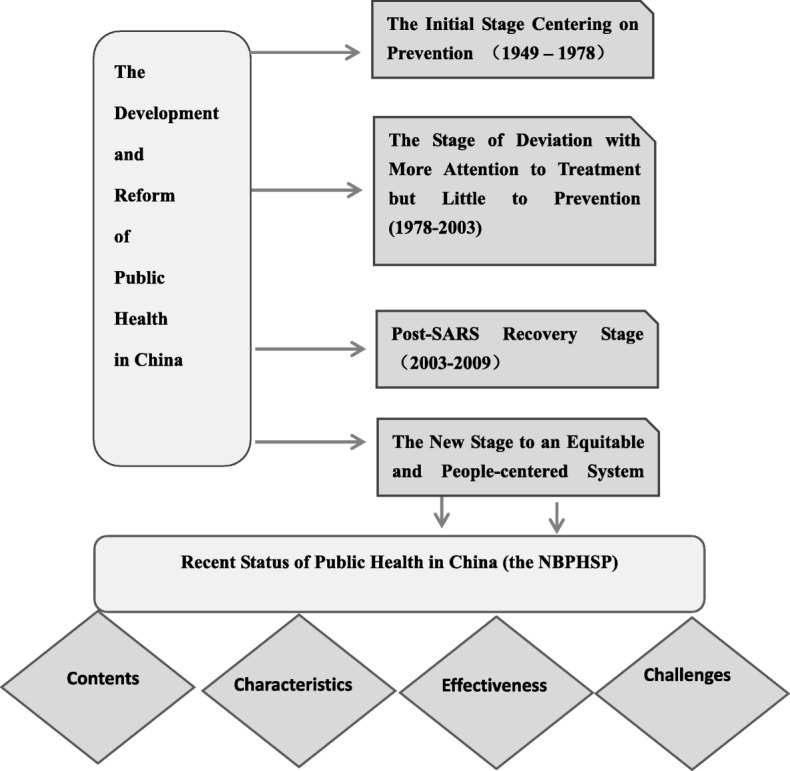
Analytic Framework on Development and Reform of China’s Public Health

According to the interviews with national-level health administrators, we identified four key stages that China’s public health experienced: the initial stage centering on prevention, the stage of deviation with more attention to treatment but little to prevention, the recovery stage after SARS, and the new stage to an equitable and people-centered system. The idea about four stages is consistent with other scholars [36, 37]. The paper takes chronologic order as its analytical framework. For each stage, the relevant socio-economic development background, the structural characteristics of the public health system, as well as the achievements and the challenges will be introduced. The descriptions on the first three stages are mainly about the chronological evolution of China’s public health sector. The fourth stage represents the latest development. This part will elaborate the NBPHSP, including its contents, characteristics and results (see Fig. 1). Restrained by the length of the paper, the study will mainly focus on disease prevention and control system which is the major system to deliver public health services to the total population and specific target groups. Moreover, it will also briefly review the evolution of health supervision system and public health emergency response system, so as to present a complete picture of China’s public health system.

## Results

### The initial stage centering on prevention (1949–1978)

Shortly after the founding of P.R.C., medical resources was in great shortage in vast rural areas, infectious diseases and endemics ran rampant, and health of Chinese residents was extremely poor. For example, the plague spread to more than 500 counties in 20 provinces (or autonomous regions); 11 million people suffered from schistosomiasis with a prevalence of more than 2 million square kilometers; patients with filariasis exceeded 30 million; the prevalence of TB was as high as 4%; and leprosy patients totaled nearly 500,000 [37]. At that time, China’s public health system was very weak. In 1949, there were only 9 maternal and child hospitals (health centers, and stations) and 11 specialized prevention and treatment centers [38].

Under such a grim situation, the Chinese government proposed guidelines for health-related work: “oriented to workers, farms and soldiers, prevention first, uniting TCM and western medicine doctors, and combining health undertakings with mass movements”, which pointed out the direction for China’s health sector. Among them, “prevention first” is the most crucial principle throughout the stage [39]. Prevention and control of infectious diseases was the core of health-related work at that time.

In order to strengthen the leadership in public health, then Ministry of Health set up Public Health Bureau specialized in epidemic prevention and related care in November 1949. The bureau was composed of Epidemic Prevention Division and Preventive Care Division, and responsible for infectious disease control, traffic quarantine, environmental health, food hygiene, school sanitation, workplace sanitation and health inspection throughout the country [37]. In 1953, the Public Health Bureau was renamed the Department of Epidemic Prevention. A vertical system nationwide for disease prevention was established from the national level down to the local level. All provinces (municipalities reporting directly to the government, and autonomous regions), prefectures and counties set up their own epidemic prevention stations. So did some industries and large factories and mines. Following the principle of “prevention first”, equipped with theories and skills of preventive medicine, those stations were responsible for disease control and surveillance, health inspection, health promotion, scientific research and training. The nationwide network covering both rural and urban areas marks the establishment of China’s initial public health system [40]. Besides the stations as the core, systems for prevention and control of endemics (such as schistosomiasis, plague, Kaschin-Beck disease, and endemic goiter), maternal and child health, and border health quarantine system were developed successively.

Meanwhile, there was another set of unique and productive working systems for public health--- the Patriotic Health Campaign. In 1952, the Central Committee of the Communist Party of China (CPC) set up the Patriotic Health Campaign Committee (the Campaign Committee) at all levels of government, a deliberative and coordinating body for health related issues. It consisted of representatives from the CPC committee, the government, the army, and the general public. Its administrative body, Office of the Campaign Committee, was established throughout the country and subject to the leadership of the government at the same level. Later, in the *Agricultural Development Outline (Draft)*, the CPC Central Committee identified killing the “four insects (namely, flies, mosquitoes, rats, and cockroaches)”, “sanitation improvement (cleaning up garbage, drinking clean water, appropriate disposal of human waste, etc.)” and “eliminating diseases” as the focuses of the Campaign [41]. Those efforts played a significant role in control of epidemics, like encephalomyelitis, malaria, measles and typhoid, in rural areas from middle and late 1960s to late 1970s [41]. Afterwards, the Campaign was transformed from a mass movement to an institutionalized government routine. It is a role model of multi-agency cooperation for health [42], and its effective mechanism of social mobilization and mass participation are regarded as successful experience of China’s public health. On July 5, 2017, the WHO presented the Chinese Government with the Outstanding Model Award for Health Governance to recognize the achievements of the Campaign [43].

With great efforts to prevent and eliminate infectious diseases and endemics, the health of Chinese people continuously improved. However, at that time, China was still an economically underdeveloped country with large population, most of who lived in rural areas with scarce medical resources. Under those circumstances, Chairman Mao Zedong proposed to “take countryside as the top health priority”. Thus, China strove to strengthen its rural public health. Human resources were mainly dependent on “barefoot doctors” (the official name is “informal rural medical workers”). They had some medical knowledge and skills. They were appointed and led by local governments, but outside of government budget payroll and without fixed salary. So, they had to support themselves by farm work in addition to delivering medical services to local people. Majority of them were either from families of doctors with expertise passing from generations to generations or intellectuals who knew a little TCM. Because they spent most of their time working barefoot in the fields, they were dubbed “barefoot doctors”. In early times, it was they who made tremendous contributions to China’s public health, as their low-cost service helped address health challenges in rural areas within a short period [44]. In terms of service delivery system, a three-tiered network composed of county, township, and village level was established. County-level health institutions took the lead, THCs were major players, and village clinics served as the foundation. The three levels were jointly responsible for preventive care, health supervision, health education and technical guidance on family planning in rural areas [45]. What’s more, they complemented and coordinated with each other in delivering curative, preventive and other health care [46]. As for health resources, the government funded infrastructure and personnel of the epidemic prevention and control institutions, and exempted them from tax. Free services were provided to control the infectious diseases threatening public health, such as universal vaccination against cowpox and BCG starting in 1950, as well as examination and treatment for schistosomiasis in 1966 [42].

Under the guidance of “prevention first”, despite the scarcity of health resources and the rampancy of infectious diseases, China’s public health sector grew rapidly in early days, by strengthening primary healthcare organizations, centering on prevention and carrying out large-scale mass movement in health. By the release of the Declaration of Alma-Ata in 1978, China’s under 5 mortality rate had dropped sharply to below 69.6‰ from 200‰ before 1949 [47]. Remarkable improvements in accessibility and equity by low-cost public health services greatly enhanced the health status of Chinese citizens in both urban and rural areas [36].

### The stage of deviation with more attention to treatment but little to prevention (1978–2003)

After reform and opening-up policy was introduced in 1978, China gradually transformed from a planning economy to a market-based one. The tendency also emerged in health sector. Government rolled back its investment, and stressed service charge as major revenue source for the operation of hospitals [42, 48]. As resources were allocated based on market mechanism, health institutions started to compete for more resources to support themselves, mainly by expanding the size, increasing the number of beds, blindly procuring high-end sophisticated equipment and devices, and paying great attention to the most profitable service items, such as some examinations, lab tests, drugs, consumables, etc. [9].

However, public health institutions were dwarfed ran into difficulties. Though disease prevention and control institutions at all levels were fully financed by the government, the fiscal resources allocated to them had relatively scaled back since 1980s [49]. In accordance with a large-scale survey at that time, 77.6% public health workers didn’t think the grassroots epidemic prevention stations had enough resources to keep functioning and cover their personnel expenses [49]. Starting from early 1980s, the government phased in self-financing policy for those stations, and allowed them to provide some charged services. So they shifted from solely government-financed institutions to ones with mixed revenue sources from both government and charged services [36] . Public grant as a share of their total expenditure declined. In a county-level station, it was only 22.4% in 1999 [49].

To make ends meet, many public health institutions began to provide more profitable charged services [46]. As many public health service providers mainly delivered charged outpatient and inpatient services, their capacity in epidemic prevention and control was waning. Meanwhile, brain drain was serious. Net staff increase was − 1.25 at county-level public health institutions and − 1.11 at township-level public health institutions, respectively, while in county hospitals it was 1.05 [49]. Public health in rural areas was on the brink of collapse. Only 1/3 public health institutions at or below county level functioned well, other 1/3 struggled to survive, and the rest 1/3 were out of operation. The epidemic control network was badly damaged [36].

Under such circumstances, *the features of China’s three-tiered service delivery network-- prevention first and coordination among each other in preventive, curative and other health care--diminished gradually (according to an interviewee from provincial CDC)*. Health providers at county, township and village level were all motivated by higher revenue and the original division of labor among the three ran into chaos. The providers usually paid much attention to treatment but little to prevention, and covered the loss from the latter with the revenue from the former [46].

In spite of the setbacks, China was still working hard to strengthen its public health system during this period. In 1986, then Ministry of Health set up the Bureau of Endemic Prevention and Control. Meanwhile, as health sector became more market-based with more market players, there was a strong demand for better health inspection and supervision [24]. In January, 2002, the Center of Disease Control and the Bureau of Health Inspection and Supervision were established. The function of health supervision was spun off from disease prevention and control system. By July, 2007, the reform on the health supervision system at national and provincial level had finished. The two arms of public health was defined more clearly. In 2002, China CDC established Center for NCD Prevention and Control to monitor and manage NCDs [25].

### Post-SARS recovery stage(2003–2009)

In 2003 when SARS epidemic broke out, the Chinese government was completely unprepared for it, and found various deficiencies in disease prevention and control, information collection, epidemic reporting, and emergency response [50]. After that, public health was once again taken seriously, and a new round of reform was immediately launched, aiming to establish a sound public health system with a public health emergency response system, a disease prevention and control system, and a health supervision system as priorities [51]. A series of response plans, laws and regulations related to public health, food safety and animal epidemic emergencies were promulgated [52].

During this period, the government increased its financial support to public health institutions. From 2003 to 2006, with financings from both national and local governments, China reinforced the disease prevention and control system and the public health emergency response system covering urban and rural areas. In 2005, vaccines covered by the National Immunization Program were completely free. In 2007, China implemented the National Expanded Program on Immunization, and free vaccines expanded from 6 items to 14 preventing 15 infectious diseases. The country eliminated smallpox and neonatal tetanus, and became a polio-free country [42]. Since 2009, basic public health services have been provided to all urban and rural residents free of charge, so that residents can enjoy equal access to those services regardless of local economic development. For the NBPHSP, service delivery cost, personnel expenditure, infrastructure investment and operating cost of specialized public health institutions were fully financed by the government budget [42].

After SARS, China established the world’s largest reporting system of infectious disease epidemics and public health emergencies. All types of health institutions at all levels, including THCs, can directly report infectious disease cases and public health emergencies to the national level. As a result, the average reporting time after detection and diagnosis by healthcare institutions reduced from 5 days to 4 h. At present, 100% disease prevention and control institutions at or above the county level, 98% of healthcare institutions at or above the county level, and 94% of primary facilities can directly report cases of statutory infectious disease on real time basis [53]. Meanwhile, surveillance of NCDs and their risk factors started, including tumor registration, death cause monitoring, NCD risk factor survey and detection of major conditions (cardiovascular) [25].

Chinese health supervision system also developed further. In 2004, then Ministry of Health formulated a document, *Several Provisions on the Development of Health Supervision System*. Provincial health supervision agencies were then established in 31 provinces (autonomous regions and municipalities directly under the central government), and more than 80% of prefectures (municipalities) and more than 50% of counties (districts) set up independent health supervision agencies [36].

**Fig. 2:**
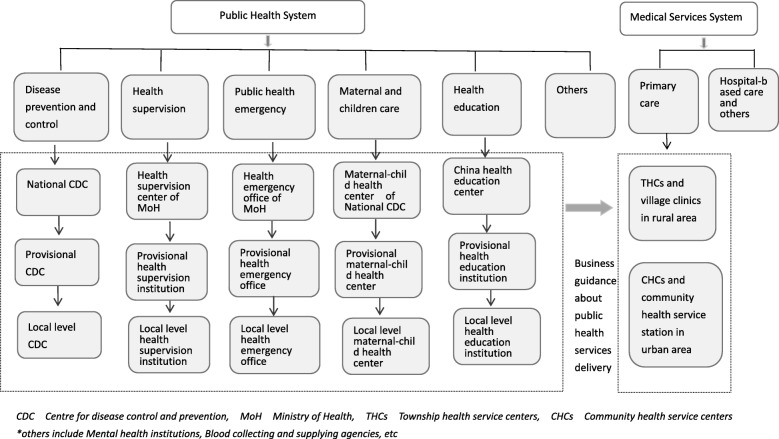
Structure of Chinese Public Health System. Source: developed by the authors

After the development during this period, a sound organization and management system of public health took shape, composed of specialized public health institutions (disease prevention and control, health education, maternal and child health, mental health, emergency response, blood supply, health supervision, family planning, etc.) for technical guidance, and community health centers (or stations), THCs, and village clinics for public health service delivery. In terms of administration, Bureau of Disease Prevention and Control, Bureau of Inspection and Supervision, Health Emergency Response Office, Department of Primary Health, Department of Maternal and Child Health, and Department of Food Safety Standards, Risk Surveillance and Assessment within the National Health and Family Planning Commission (the predecessor of current NHC) were responsible for public health administration at the national level, and competent units within provincial, municipal, or county-level health authorities were accountable for local public health management [42]. The public health architecture is shown in Fig. 2.

Outbreak of SARS was a public health disaster for China, but it was also an opportunity to revitalize the system and motivate Chinese government to renewed its commitment to public health and people’s health. However, there remained a number of long-standing drawbacks. First, as an interviewee from the Bureau of Disease Prevention and Control, NHC, said, *medical care system (hospitals at all levels) and public health system were still two separate silos*. The disease prevention and control institutions were fully and solely financed by government budget, while the medical institutions were funded by government budget and markup on drugs (canceled in 2017) and service charges. The average income in general hospitals had always been higher than that in disease control institutions, leading to insufficient incentives for professionals in the latter and even brain drain. It was particularly difficult to retain those with clinical background. Without enough competent professionals, in case of infectious disease emergencies, public health institutions might just wring their hands in diagnosis and disposal [54]. Second, at that time, public health services didn’t cover the total population. Most services were about prevention and control of infectious diseases and endemics, and few resources were allocated to health education, health management, and NCD control. According to an interviewee from national-level CDC, *ordinary citizens had no opportunity or ability to seek public health services actively. Nor did they have the awareness.* Third, there was a big gap in people’s health status and accessibility to public health services between rural and urban areas and among regions [55]. The interviewees from provincial level and below also agreed those ideas.

### The new stage to an equitable and people-centered system (2009 up to now)

At this stage, the focus of China’s health system has shifted from framework development to equity and people-centered model. In 2007, the report of the 17th CPC Party Congress stated that public health should remain public goods, and public health service and essential health care should be effective, accessible and affordable, which identifies the direction of China’s health system. After the 18th CPC Party Congress, China has been striving for a well-off society by 2020 and health is part of it. On many occasions, Xi Jinping stresses that there is no prosperity without healthy population [56]. Healthy China has become a significant national strategy. According to the Planning Outline of Healthy China 2030, everyone will have access to comprehensive healthcare services covering the whole life cycle by 2030, average life expectancy will be 79, and major health indicators on par with high-income countries. Evidence shows that public health can contribute greatly to better health indicators [3]. For China, a populous developing country with dual disease burden, public health is indispensable to healthy population and healthy China.

In 2009, the Chinese government launched a new round of healthcare reform and identified four pillars of China’s health system, and equitable and accessible public health service system is one of these four pillars. Later, the goal of “equalization of basic public health services” was set, and the “National Basic Public Health Service Program” was launched [57].

**Fig. 3:**
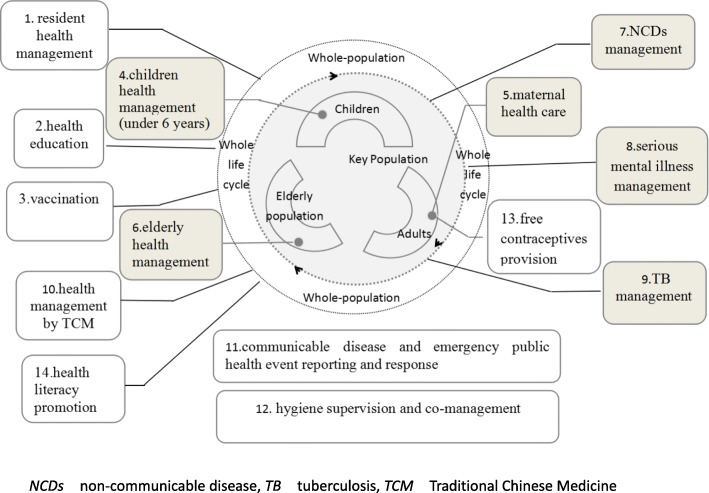
Service package of the Program covering the whole life cycle and all the population. Source: National Basic Public Service Specifications 2017

The people-centered program provides both population-wide interventions and targeted services specifically for pregnant and postnatal women, children, the elderly and those with NCDs or TB, to meet the needs of total population in the whole life cycle (See Fig. 3).

Moreover, reporting and handling of infectious diseases and public health emergencies as well as health supervision is also included, which makes the Program comprehensive and systemic. It is completely funded by the government, and directly benefits urban and rural residents. In urban areas, the relevant services are delivered by the community health centers (CHCs) or stations, and in rural areas by THCs and village clinics. The stations and clinics provide some services as appropriate under the technical management of CHCs and the THCs respectively [58]. To better deliver and manage the basic public health services, the Chinese government issued three editions of *National Basic Public Service Specifications* in 2009, 2011, and 2017 respectively. The service package expanded from 41 items in 9 categories in 2009 to 55 items in 14 categories in 2017 (See Table 1 for details).

**Table 1:** Service package of the program (2017)

No.	Category	Target population	Service item and its content
1	Residents health records	residents in the catchment, including the non-registered people (those without local registered permanent residence) living there over half year	1. establishing health record
			2. maintaining and managing health record
2	Health education	residents in the catchment	1. providing health education materials
			2. setting health education bulletin board
			3. health consultation service for the general public
			4. holding lectures on health
			5. personalized health education to individuals
3	Vaccination	children aged 0~6 and other priority group in the catchment	1. vaccination management
			2. vaccinating the population
			3. handling suspected adverse events after vaccination
4	Health management of children aged 0~6	children aged 0~6 in the catchment	1. postpartum newborn home visit
			2. health management of 1-month-old baby
			3. infant health management
			4. health management of children aged 0~6
5	Maternal health care	pregnant or postnatal women in the catchment	1. health management in early pregnancy
			2. health management in mid pregnancy
			3. health management in late pregnancy
			4. postnatal visit
			5. 6-week postpartum checkup
6	Elderly health management	residents at or above 65 in the catchment	1. lifestyle and health status evaluation
			2. physical examination
			3. auxiliary examination
			4. health guidance
7	NCD management (hypertension)	primary hypertensive patients at or above 35 in the catchment	1. screen
			2. follow-up evaluation and classified intervention
			3. health checkup
	NCD management (type 2 diabetes)	type 2 diabetic patients at or above 35 in the catchment	1.screen
			2. follow-up evaluation and classified intervention
			3. health checkup
8	Serious mental illness management	Patients diagnosed with serious mental health conditions and living at home in the catchment	1. patient information management
			2. follow-up evaluation and classified intervention
			3. health checkup
9	TB management	Patients diagnosed with TB and living at home in the catchment	1. reference to the people suspected symptoms
			2. follow-up and medication management (for patients with MDR TB) and guidance
			3. health checkup
10	Reporting of and response to infectious diseases and public health emergencies	population in the catchment	1. management of infectious diseases and public health emergencies
			2. detection and registration of infectious diseases and public health emergencies
			3. reporting the information related to infectious diseases and public health emergencies
			4. handling infectious diseases and public health emergencies
11	Health management by TCM	residents at or above 65 and children aged 0~6 in the catchment	1. identifying the constitution of the elderly by TCM
			2. care of the children by TCM
12	Health inspection and supervision	residents in the catchment	1. report on food safety
			2. report on occupational health consultation and guidance as well as family planning
			3. inspection on safety of drinking water
			4. school-based health services
			5. report on illegal practice and illegal blood banks
13	Free contraceptives	residents in the catchment	1. provincial health and family planning authorities procure contraceptives according to laws
			2. Provincial, municipal and county-level health and family planning authorities store, distribute and allocate the contraceptives.
14	Health promotion	residents in the catchment	1. building Health Promotion County (District)
			2. popularization of health science
			3. developing health promotion hospital and tobacco control clinic
			4. monitoring health literacy and tobacco use prevalence
			5. 12,320 hotline service
			6. health education on key areas and major conditions to key groups

*NCDs* Non-communicable diseases, *TB* Tuberculosis, *MDR-TB* Multi-drug resistant tuberculosis, *TCM* Traditional Chinese Medicine

Source: *National Basic Public Service Specifications 2017 (the 3rd edition)*

**Fig. 4:**
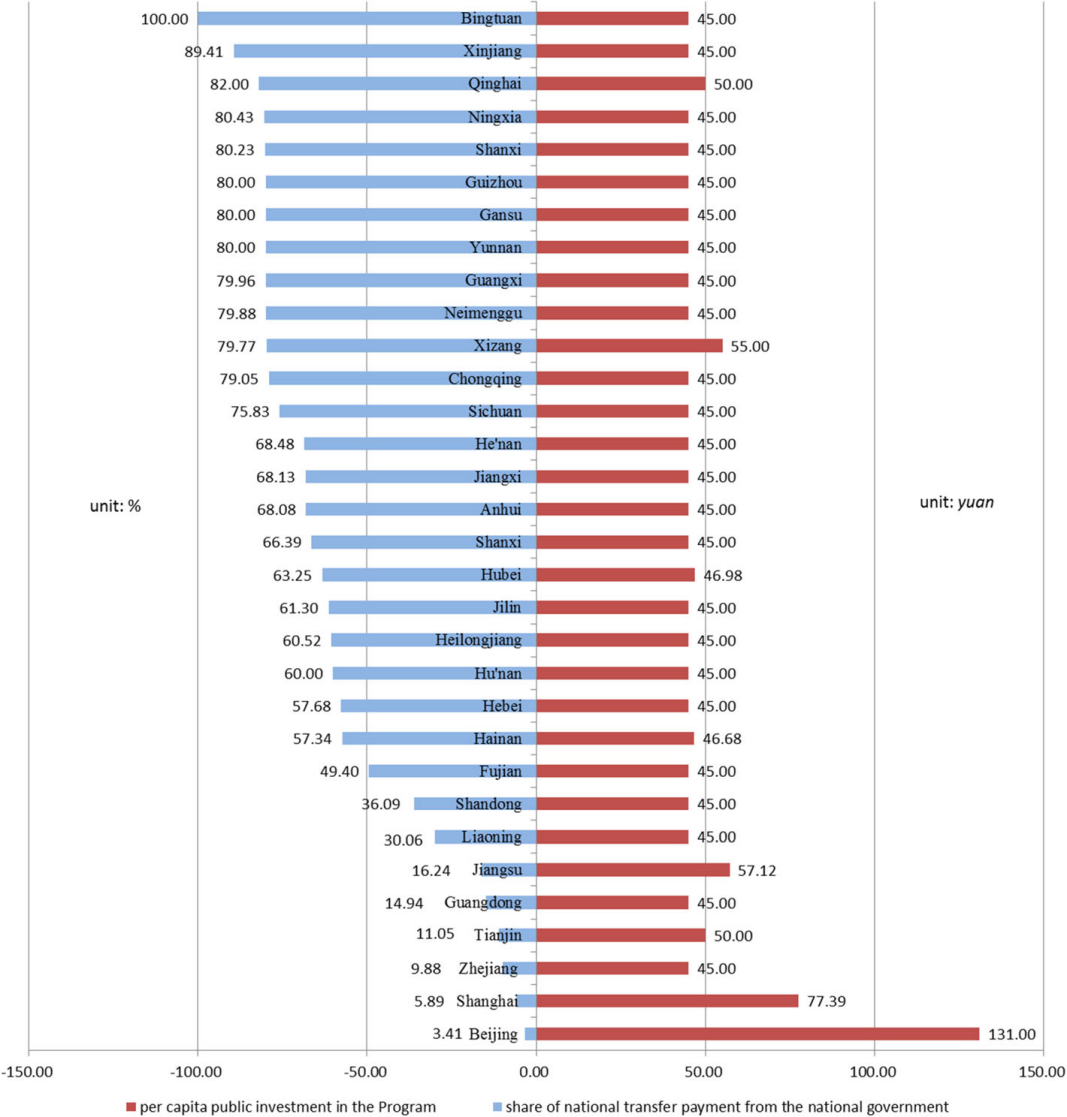
Per Capita Public Expenditure on the Program and the Share of Transfer Payment from the National Government by Provinces in 2016. Source: National Survey on Primary Health Facilities 2016, by the Department of Primary Health, NHC, China

Due to the extremely uneven economic development in China, fiscal situation of local governments varies. To ensure the financing equity in basic public health services, the national government set a minimum standard for per capita public investment in the Program. To be specific, it was 45 Yuan in 2016, 50 Yuan in 2017 and 55 Yuan in 2018 (1 US$ = 6.71 RMB yuan, 25 Feb., 2019). In underdeveloped areas, it is mainly financed by the national government [55]. In 2016, 70% of the financing came from the national government in 13 provinces (autonomous regions and municipalities directly under the central government) out of the 32.^1^ Meanwhile, from efficiency perspective, provincial and municipal governments are allowed to raise the standard and expand service package to maximize the effects of the NBPHSP [59]. In 8 provinces, the per capita financing is higher than the national standard, namely, Qinghai, Tibet, Hubei, Jiangsu, Tianjin, Shanghai, and Beijing. In Beijing and Shanghai, it was even 136 yuan and 77 yuan respectively. See Fig. 4 for details.

In the nine years since the launch of the Program, tremendous efforts have been made to cover all of the target population. Some services are for the general population while others target some specific groups, such as children aged 0~6, pregnant and postnatal women, the elderly, hypertensive patients and diabetic patients. Most of interviewees from national level and below argue that *the implementation of basic public health services has shown positive results to some extent, and it can be seen that the government pay a significant attention to management of chronic diseases.* We selected 12 indicators to evaluate the implementation of the Program, namely, coverage of health record, coverage of electronic health record (EHR), utilization rate of health record, rate of postpartum newborn home visit, coverage of health management among children aged 0~6, rate of registration in early pregnancy, coverage of postnatal visit, coverage of health management among the elderly, percentage of hypertensive patients under standardized management, percentage of type 2 diabetic patients under standardized management, coverage of health management by TCM among the elderly, and coverage of health management by TCM among the children aged 0~6. The detailed definitions are shown in Table 2.

**Table 2:** Some indicators of the program and their definition

Target Population	Name of the Indicator	Definition	Notes
General population	coverage of health record	residents with up-to-standard health record in the catchment / total residents in the catchment× 100%	Up-to-standard health record refers to the record with completely filled cover page and individual information form. Individual information of children aged 0~6, is filled in “postpartum newborn home visit form”, rather than the individual information form. The numerator should exclude those who are dead, move out or are not contactable by all means.
	coverage of electronic health record (EHR)	residents with up-to-standard EHR in the catchment / total residents in the catchment×100%	Up-to-standard EHR refers to the record with complete cover page and individual information form in the electronic health record management system.
	utilization rate of health record	number of those with dynamic updated information among the randomly sampled records/ total records sampled × 100%	Updated information refers to the information on the utilization of medical services and other health services compliant with service specifications within 1 year.
Specific population	rate of postpartum newborn home visit	number of newborn babies in the catchment who receive postpartum home visit or health management of 1-month-old infant for at least once in the year / total live births in the catchment in the year ×100%	Live births refer to the babies in the catchment born at and after 28 weeks’ gestation, or with birth weight of 1000 g and above, who shows any sign of life, including respiration, heartbeat, umbilical pulsation, or movement of voluntary muscles, from the beginning of the year to the data collection date.
	coverage of health management among children aged 0~6	number of children aged 0~6 in the catchment who receive one or more follow-ups in the year/ total children aged 0~6 in the catchment in the year ×100%	Children aged 0~6 refer to the children in the catchment who have not got to their 7th birthday as of the data collection date. If real-time data are not available, the number of children aged 0~6 at the end of the previous year can be taken as proxy.
	rate of registration in early pregnancy	number of pregnant women in the catchment who get registered and have 1st prenatal checkup in a hospital before 13 weeks’ pregnancy in the year/ number of live births in the catchment in the year ×100%	The number of pregnant women in the catchment who get registered and have the 1st prenatal checkup in a hospital before 13 weeks’ gestation refers to the number of pregnant women with live births, who get registered and have the 1st prenatal checkup in a hospital before the 13th week of pregnancy (12 weeks plus 6 days) from the beginning of the year to the date of data collection.
	coverage of postnatal visit	number of postnatal women in the catchment who receive postnatal visit within 28 days after discharge from the hospital/ live births in the catchment ×100%	The number of postnatal women in the catchment who receive postnatal visit within 28 days after discharge from the hospital is the number of postnatal women in the catchment who receive postnatal visit within 28 days after discharge from the hospital from the beginning of the year to the date of data collection.
	coverage of health management among the elderly	number of elderly residents in the catchment who receive health management services / total residents aged 65 or above in the catchment ×100%	The number of elderly residents who receive health management services refers to the number of permanent residents aged 65 or above who receive all of the four health management services, namely, establishment of health record, physical examination, health guidance and complete physical examination form, from the beginning of the year to the date of data collection.
	percentage of hypertensive patients under standardized management	number of hypertensive patients in the catchment under management up to the standards of the specifications / total hypertensive patients under management in the catchment in the year ×100%	A patient is regarded as under standardized management, if in the Nth quarter of the year, the patient received N or more follow-ups from the beginning of the year to the date of the data collection report, or if the patient received 4 follow-ups and 1 physical examination annually in the previous two years. The denominator is the number of hypertensive patients in the catchment receiving at least one follow-up from the beginning of the year to the date of data collection.
	percentage of type 2 diabetic patients under standardized management	number of type 2 diabetic patients in the catchment under management up to the standards of the specifications / total type 2 diabetic patients under management in the catchment in the year×100%	The definition of standardized management is the same as that for hypertension management.
	coverage of health management by TCM among the elderly	number of the elderly residents aged 65 or above in the catchment who receive TCM health management services in the year/ total residents aged 65 or above in the catchment in the year ×100%	TCM health management services refer to constitution identification by TCM, health guidance, and complete service record form in the health record of the resident, from the beginning of the year to date of data collection.
	coverage of health management by TCM among children aged 0–36 months	number of children aged 0–36 months in the catchment receiving month-age-based TCM health management in the year/ total children aged 0–36 months in the catchment who should be under TCM management in the year × 100%	

*EHR* Coverage of electronic health record,, *TCM* Traditional Chinese Medicine

Source: *National Basic Public Service Specifications 2017 (the 3rd edition)*

**Fig. 5:**
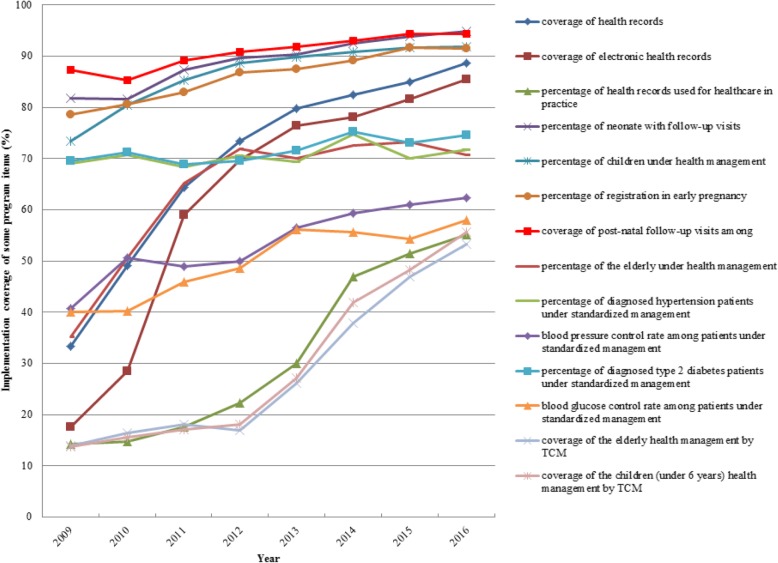
Some Indicators about the Implementation of the Program 2009–2016. Source: National Survey on Primary Health Facilities 2016, by the Department of Primary Health, NHC

For the general population, the first and most basic service provided by the Program is to establish health record. *Unlike citizens in developed countries with mature health record system, Chinese residents rarely had their own health records before, Establishing health records can provide better public health services (*an interviewee from national-level CDC*).* Good health records can help doctors get accurate and critical information about their patients very soon, such as patients’ condition, medical history, family history, and personal lifestyle, and provides evidence for healthcare services throughout the life cycle [60]. By 2016, coverage of health record, hard-copy or electronic, had been over 85%. Utilization rate of the records had increased from less than 15 to 55% (as shown in Fig. 5).

Services targeting specific groups have grown stably in the past 9 years. Rate of postpartum newborn home visit, percentage of children under health management, rate of registration in early pregnancy, and coverage of postnatal follow-up had all exceeded 90%. Coverage of health management among the elderly was more than 70%. For those with NCDs, such as hypertension and type 2 Diabetes, percentage of patients under standardized management stood at 70%. Control rates of blood pressure and blood glucose both reached 60%. Health management by TCM gained its momentum. The coverage of health management by TCM among children aged 0–36 months was more than 50% and so was that of the elderly. In a nutshell, the Program provides comprehensive public health services to hundreds of million Chinese people [61] (Fig. 5).

Among the services targeting to specific groups, neonatal and maternal care is the top priority. Compared with other countries, the infant mortality rate (IMR) in China has decreased year by year from 2009 to 2016, down by 8.22% annually, ranking the 4th globally (*n* = 193). The maternal mortality rate(MMR) dropped by 5.52% annually from 2009 to 2015, ranking the 17th in the world (*n* = 183). The data of some countries are shown in Table 3 and Table 4. It is clear that since the launch of the Program in 2009, the decline in IMR and MMR in China has been among the biggest in the world. In absolute terms, China still lags behind high-income countries such as Japan, South Korea, and the United Kingdom. However, as a developing country, China has succeeded in controlling the two rates below the global average and even below the average of upper middle-income countries. It is fair to say that its effort to control IMR and MMR is of good quality and high efficiency, and the Program is an essential part of it.

**Table 3:** Infant mortality rate in selected countries

Country	Income level	Annual reduction 2009–2016	Ranking	Absolute value in 2016(‰)	Ranking
Kazakhstan	upper middle-income country	9.96%	1	5.9	67
Montenegro	upper middle-income country	9.68%	2	2.4	27
Latvia	high-income country	8.58%	3	2.4	27
China	upper middle-income country	8.22%	4	5.1	59
Estonia	high-income country	7.81%	5	1.3	7
Japan	high-income country	3.92%	37	0.9	2
India	lower middle- income country	3.79%	43	25.4	163
ROK	high-income country	2.53%	100	1.5	10
U.K.	high-income country	2.47%	103	2.6	33
U.S.	high-income country	1.79%	139	3.7	44
Germany	high-income country	0.60%	174	2.3	25
World average	–	2.93%	–	18.6	–
high-income countries	–	1.76%	–	3	–
upper middle-income countries	–	4.85%	–	7.2	–
middle-income countries	–	3.17%	–	18.6	–
lower middle-income countries	–	2.91%	–	24.8	–
Low-income countries	–	2.75%	–	26.9	–

Source: *WDI* The World Bank Databank (2016)

**Table 4:** Maternal mortality rate in selected countries

Country	Income level	Annual reduction 2009–2015	ranking	Absolute value in 2015(1/100,000)	ranking
Kazakhstan	upper middle-income country	10.74%	1	12	43
Turkey	upper middle-income country	8.29%	2	16	51
East Timor	lower middle-income country	7.80%	3	215	134
Laos	lower middle-income country	7.57%	4	197	132
Ethiopia	low income country	7.57%	5	353	149
China	upper middle-income country	5.52%	17	27	66
ROK	high-income country	4.97%	24	11	39
India	lower middle-income country	4.19%	43	174	128
Japan	high-income country	2.78%	79	5	11
Germany	high-income country	2.38%	98	6	16
U.K.	high-income country	1.67%	129	9	30
U.S.	high-income country	1.11%	146	14	46
world average	–	2.66%	–	216	–
high-income countries	–	1.52%	–	10	–
upper middle-income countries	–	3.88%	–	41	–
middle-income countries	–	2.61%	–	180	–
lower middle-income countries	–	2.57%	–	254	–
low-income countries	–	3.49%	–	496	–

Source: *WDI* The World Bank Databank(2015)

At present the Program is pushed ahead with stably, but still faces up with many challenges.

#### Quality of the basic public health services is yet to be improved

First, as some primary health workers lack knowledge or awareness, service quality is to be enhanced. For example, NCDs management is not up to the standard or clinical decisions are not correct sometimes. In Guangxi Province, Tan et al. conducted a survey about how well health workers knew the policies related to equalization of basic public health service. It found that, only 45.12% of respondents knew the related policies well and health workers paid little attention paid to those policies [62].

Second, the service package of the Program cannot meet the actual needs of the general public. It is greatly associated with the fact that service package is set based on how much public financing is available [63]. *With better health literacy, health needs of Chinese citizens are growing and getting diversified, while the existing package fails to catch up the changes (according to a public health doctor of a THC*). Moreover, public health priorities are dynamic, which requires timely updating and adjustment of the service package [64].

In addition, the Program covers so many services and such a huge number of target population, that primary health workers can only manage to hit the set target but compromise on quality. *A village doctor said in an interview:* “*We spend most of our time filling the forms and keeping the records. We are busy all day long to pass the performance evaluation. I guess we don*’*t get the exact point and purpose of public health*”. Quality and precision of services sometimes is far from the standards set in the *Basic Public Health Service Specification*. For example, some follow-ups do not comply with the *Specification*. Sometimes, providers just take the service as a matter of formality, or even forge the service records [63]. Latest studies indicates that quality of care, not just accessibility, is the key determinant to improve population health and reduce disease burden [65].

#### Poor integration among service items and in service delivery system lowers the efficiency

Poor integration among the service items impacts the efficiency of the system. There are so many service items in the package but little integration among them. For example, besides population-wide health record, separate records are required for maternal and child health as well as elderly health management. Similarly, NCD cases are managed by conditions in an isolated way. All of these involve much unnecessary duplication in efforts and systems. Meanwhile, essential health care should highlight the principle of integration of preventive and curative care. But China’s basic public health services and essential medical care are not well connected. Take NCD management as an example. S*ervices by GPs are poorly integrated with those provided by public health workers* (according to an interviewee from county-level health authorities). Better integration will boost the efficiency of the system. South Africa made impressive progress in primary care largely because of its attention to integrated care [66]. Ngazi also appealed that UHC requires a more integrated health system including public health [67].

Furthermore, *IT system is fragmented and lags behind. In most provinces, IT systems in primary healthcare facilities are backward and not compatible and connected with each other* (according to an interviewee from the Division of Primary Health in provincial health authorities). Li’s study shows that systems of the primary institutions with electronic medical record are developed independently by over 80 IT providers with little connectivity and interoperability. Poor integration among IT systems hinders information sharing across institutions and regions, and impacts the outcomes of the Program [21]. For instance, although EHR covers more and more population, their utilization in practice is unsatisfactory. Moreover, the EHR system is not linked with the health providers’ IT system. The fragmented IT systems are another barrier of integration of preventive and curative care. For another instance, China used various tracking systems to monitor environmental public health, however, the systems were isolated from each other due to the lack of information sharing among different departments, then leading to knowledge gap about the environment and public health [68].

#### Well-trained health professionals are in shortage

The third challenge is insufficient well-trained primary health workers. They are the major implementers of the Program. But both the quantity and the structure of the health workers are yet to be improved [21]. Data of 2013 showed that only less than 36% health professionals (21% nurses) worked in THCs and CHCs [69]. GP (5.2% of doctors) is one of the professionals in most shortage. In January, 2018, GPs per 10,000 people in China was 1.5, still far away from the target for 2020, 2–3 GPs per 10,000 people [70]. The professionals do not increase proportionately with the expansion of service package. *The challenge was aggravated by poor integration among the service items* (according to an interviewee from county-level health authorities).

In practice, it means more and more workload on the existing health workers in the institutions. This study conducted a survey among primary health workers in 3 provinces. The result shows that 27.1% thought that after the introduction of the Program, workload increased to an unbearable level. Shi indicates that 4307 out of 10,626 (41%) primary physicians felt extremely tired [71].

Moreover, primary health workers are often less knowledgeable with lower education level and professional title [72]. Among staff in CHCs and THCs, those with associate degree or below accounted for 71.9 and 91.3% respectively. Village doctors were even more poorly educated.^2^ 21% of primary health workers are not licensed practitioner or licensed assistant practitioners [21].

Lack of human resources and inadequate service capacity has greatly impacted the implementation of the Program. A qualitative study in Beijing finds that GPs are too insufficient in terms of both quantity and technical skills to deliver adequate basic public health services to the population in the community [73]. In some places, a provider struggle to reach the set target of service delivery, and ignore the challenges like understaffing and poor capability, overlooks the effectiveness of service provision, and even falsifies related records.

#### Other sectors are not fully engaged

Fourthly, China’s basic public health service delivery system is still an isolated island. The services are mainly delivered by health institutions at present. However, health not only refers to physical health, but also includes mental and psychological health, and involves social, environmental and ethical aspects. So it also depends on factors other than health care. Non-health sectors, such as transportation, agriculture, land use, real estate, public security, and education systems, can affect people’ s health as well [74]. Given the complexity of the issue, it is imperative that government agencies and civil society should work together [75]. There are many effective intersectoral heath promotion programs such as Finland Mental Health Improvement, The Public Health Agency of Canada’s Innovation Strategy, Health Promotion Switzerland, Thai Health Promotion Foundation [76].

In recent years, “health in all policies” (HiAP) has become an important guideline for China‘s efforts in health [77]. HiAP is also described as an essential component of primary health care [78]. Nevertheless, there are still some problems about how to realize HiAP in China [79]. The NBPHSP has recognized the negative impact of unhealthy lifestyle. But it is not enough to just change individuals’ lifestyle. More attention should be paid to related social and environmental factors. For example, people can have good lifestyle and dietary structure, but cannot avoid the negative impacts of inhaling polluted air during outdoor exercises or intaking antibiotics, steroids and pesticide residues in diet. The Program should seize the opportunity to work with more partners, especially the players in non-health sectors, to address the elements at policy and structural level, not just at individual level [77].

## Discussion

China’s public health has developed for 7 decades since the founding of P.R.C. Looking back, there was unique Chinese wisdom and remarkable achievements as well as twists and turns on the journey of reform. In the past 70 years, China has made great strides in providing equitable and accessible public health services to its citizens, and built up a well-established service delivery system [64]. As a result, the health status of Chinese people has been enhance significantly [25] since 1949, and public health has contributed 77.9% to the increase of life expectancy [19]. It is similar to the situation in other countries. In the twentieth century, life expectancy in U.S. rose 30 years, and 25 of it was attributed to public health [80].

In early times, China’s public health system successfully controlled the infectious diseases mainly because of: (1) The prevention-first and preemptive approach. During that period, guidelines and policies, resource allocation, as well as organizational structure of the health sector centered around prevention and control of infectious diseases. That was aligned with the spectrum of disease at that time, therefore led to outstanding outcomes; (2) Flexible structure of the system. For example, to fill the huge gap of health workforce at that time, a great amount of barefoot doctors came to the fore. They were both farmers and PHC personnel. Their income came from their farming work and payment from village collective economy for their public health services [42]. Therefore service cost was reduced significantly. Besides, their good knowledge on local environment and local people in the catchment was helpful to provide effective public health services. Thus, they made remarkable contribution to the progress in China’s public health; (3) The three-tiered service delivery network, and collaboration within the network and with non-health sectors. The three levels were complementary and coordinated in prevention, treatment and other care, and the Patriotic Health Campaign Committee (the Campaign Committee) was a good example of the multi-agency collaboration, and in line with “HiAP” far before the principle was brought forward; and (4)Innovative mechanism of mass mobilization and society participation. For instance, in addition to its administrative system to coordinate different agencies, the Patriotic Health Campaign established some civil societies at grassroots level so that every household was mobilized to implement the Campaign and the whole society participated it effectively [81].

China’s market-oriented financing reform initiated in the late 1970s created both opportunities and challenges for the health system [42]. It mobilized more resources from users of health services and improved working conditions, while it led to a dramatic reduction in government spending on health. The capacity of public health institutions in epidemic prevention and control waned because more and more public health service preferred to providing profitable charged services including outpatient and inpatient services. Especially, public health in rural areas was on the brink of collapse at that time.

Despite twists and turns, China’s public health has always been resilient. System resilience is defined as “the capacity of a system to absorb disturbance and reorganize while undergoing change so as to still retain essentially the same function, structure, identity, and feedbacks” [82]. The public health system in China was impacted badly by the market-oriented healthcare reform in the 1980s, but got back to the right track after the SARS outbreak. In many countries, public health crisis is an external driver to improve their system. From nineteenth century to early twentieth century, the rampant cholera outbreak was the main trigger for U.K. to promote its public health [83]. In U.S., due to the September 11th Attacks and the Anthrax Attacks, its conventional public health system, in which federal-state-local structure was loosely coordinated, was fundamentally reformed into a new three-tiered system composed of (federal) CDC, regional/state HRSA (Health Resources and Services Administration) and local MMRS (Metropolitan Medical Response System) [84]. The new system also stresses inter-agency collaboration and international cooperation for a strong public health net [84]. According to a study in 2003 by Hong Kong-based Political and Economic Risk Consultancy (PERC), U.S. ranks the first in terms of capacity of responding to public health emergencies.

We argue that the renewed attention to public health in China was triggered by the SARS outbreak. But the sustained progress is driven by the government’s commitment to social development and people’s livelihoods, and backed by robust economic growth and strong government leadership. After SARS crisis, government’s role and responsibilities in the health sector were further clarified, and the growth rate of government spending on health was required to be higher than the growth rate of government spending [42]. For example, per capita expenditure on NBPHSP increased from 15 yuan in 2009 to 55 yuan in 2018. This period also registered rapid economic growth and great improvement of peoples living standard in China. Jakovljevic indicated that it was the significant improvement in people’s living standard and purchasing power that provided momentum for emerging countries like BRICs (Brazil, Russian, India, China) members to rise their investment in health care far more than other countries or areas worldwide [85]. Rancic also argued that spending on health would increase as countries become richer [86]. Government investments in health and well-being of the citizens are more challenging for most Balkan countries as they were impacted by the global economic crisis in the last decade and prior history of civil war for most of them [86].

Moreover, rapid development of China’s health sector is also associated with the strong leadership of the government. A study published in China’s most authoritative journal of social sciences, *China Social Science*, argued that the Chinese state was powerful, and the Communist Party of China (CPC) was the center of the power [87]. Most major strategies and guidelines in health sector were proposed at CPC Congress, and their implementation was also followed the “public policy implementation mechanism with Chinese characteristics under the leadership of the CPC” [87]. The famous American Sinologist, Prasenjit Duara, pointed out that China’s success to much degree lies in the strong party organization, which is deeply rooted in Chinese urban and rural areas [88]. The party and the state have sufficient power of mobilization. In a word, it is fair to say that this period was the golden time for the development and construction of public health institutions [42]. For instance, the public health emergency response system was developed from scratch. Meanwhile, the conventional disease prevention and control system and the health supervision system also went from strength to strength rapidly. Zhang had a similar observation on transition trajectory of China’s rural health system [89].

In 2010s, Chinese government pays unprecedented attention to health sector, presses ahead with new round of healthcare reform, and formulates the strategies of “Healthy China” and “no well-off society without healthy population”. Among BRICs countries, China registers the most rapid development in health system and is the most significant member in terms of global outreach [85]. China’s share of nominal total health expenditure (THE) composition in BRICs rose from 29% in 1993 to 52% in 2012, gradually achieving a dominant position from year to year [90], and representing the largest share of total THE of BRICs. All of these bring a new wave of opportunities to public health in China. At this stage, the goal is to make the system more equitable and people-centered [21]. The NBPHSP remains the priority. It is designed to provide rural and urban residents with free basic public health services covering total population throughout the whole life cycle, which increases the accessibility and affordability of basic public health services. Compared with Brazil and India, rural Chinese have much higher gains in equity of access to health care in China [85], although all BRICs countries have very uneven population distribution with exceptionally large rural areas [90]. However, there are challenges in NBPHSP implementation, including concerns on quality of services, the package that is not updated timely, poor integration of the system and inadequate human resources [64].

In China, quality concerns to a large extent are attributable to insufficient per capita health expenditure. BRICs’ joint share of global health spending is far less than that of OECD [90]. And among BRICs members, per capita health expenditure in Russia and Brazilian exceeds that in China three times and more than twice respectively [90], which may indicate that Chinese health reform still has a long way to go. However, some OECD countries suffer from surge in health expenditure with few marginal health gains. China needs to avoid it, although its per capita health spending is still relatively low [91].

Integration of health systems is the direction of future efforts in the world. The United Nations (UN) Sustainable Development Goals (SDGs) highlights organic connections and systematic approach among a variety of health factors, and enhancing overall health system is more important in the SDG era [92]. However, China’s public health service system is still facing insufficient integration problems such as poor service items integration, insufficient intersetoral actions, isolated IT system and so on. The effectiveness of cooperation mechanisms on health issues among different sectors depends heavily on factors such as organizational structure, management, culture, and trust [76]. We argue that ever effective coordinate mechanisms such as the Patriotic Health Campaign Committee in China can be further applied to cope with emerging public health challenges such as ageing and NCDs.

The lack of health human resources at the grass-roots level, especially in rural areas, is an important problem that China and other emerging developing countries are facing [90]. Doctors and nurses are reluctant to be employed by primary health facilities most of which are located in the countryside. It is an obstacle to develop sufficient and effective public health workforce [90].

From global perspective, most countries are in the transformation of public health landscape, due to common emerging challenges. The development and reform of public health in China needs to be further deepened. Firstly, accelerated aging population is placing a number of countries in a substantial disadvantage position in healthcare reforms [93]. Developing countries are experiencing much more rapid aging process than rich countries, and China is the fastest one in the upcoming decades [90]. This is a serious potential risk to the financial sustainability of China’s health sector in broader sense [85]. Furthermore, lower fertility willingness may exacerbate the risk. It is similar to the situation in the Next Eleven (Next-11) countries where healthcare expenditure goes up dramatically, due to higher proportion of the elderly and lower fertility rates [94]. Secondly, NCDs are recognized as the key health challenge worldwide [95], and are already China’s number one health threat [91]. Unlike infectious diseases which have relatively short acute phase and take less time to cure, NCDs will bring massive and long-term burden for both patients and the society [95]. Moreover, the prevalence of NCDs among the elderly is disproportionately high, and some of them often have more than one NCDs [76]. Emerging NCDs burden coupled with aging population means that sustainability challenge in public health system will be very serious, even in the richest OECD countries [76]. Thirdly, social and economic transformation have accelerated urbanization and changes in life style, leading to many risk factors such as obesity, sedentary lifestyles, stress, tobacco/alcohol/other substances abuse, and exposure to pollution [95]. The incidence of NCDs is also rising due to these individual or environmental factors. Fourthly, globalization accelerates the spread of infectious diseases, imposing challenges to public health. Many countries including China face the dual burden of NCDs and infectious diseases at the same time.

In a nutshell, the evolution and reform of China’s public health is based on its national condition. During the process, China accumulates rich experience but also faces many common worldwide challenges which may be even more pronounced in China.

However, it is expected that government’s continuous attention to health sector and its stable macro environment will be greatly helpful to address those challenges. Getting this development and reform right is important to China’s social and economic development in future, and we believe that China’s experience in public health may provide many lessons for other countries.

## Conclusion

During the past seven decades, China has made impressive strides in the development of public health system, despite experiencing twist and turns. Based on the analysis above, the study suggests:

(1) Strengthening the public health service delivery system, focusing on prevention and preemptive control of diseases, and highlighting public health functionality of rural and urban primary health facilities;

(2) Empowering community and the general population by setting up multi-department coordination mechanism for social mobilization and participation based on the experience of Patriotic Health Campaign; and.
(3) continuously improving the basic public health services, including higher quality of the services, better monitoring and in-flight adjustment of the Program, efficient and relevant training for more highly-skillful professionals, and more integrated IT systems, so that everyone has equitable access to quality basic public health services.

## Data Availability

Data can be made available by request.

## Abbreviations

CHCs: Community Health centers
CPC: The Central Committee of Communist Party of China
GPs: General practitioners
HiAP: Health in all policies
NBPHSP: National Basic Public Health Service Program
NCDs: Non-communicable diseases
NHC: National Health Commission
SARS: Severe Acute Respiratory Syndromes
SDGs: Sustainable Development Goals
TB: Tuberculosis
THCs: Township health centers
THE: Total health expenditure

## Footnotes

1 The data of “Xinjiang Production and Construction Corp”was collected separately from those of Xinjiang Vigor Autonomous Region. the autonomous region. “Xinjiang Production and Construction Corp” is an important component of Xinjiang Vigor Autonomous Region and responsible for both national government and the autonomous region. The Corp has its own administrative and judicial system.

2 Calculated with the data from China’s Statistic Yearbook of Health and Family Planning 2017.
